# Host-virus interaction: a new role for microRNAs

**DOI:** 10.1186/1742-4690-3-68

**Published:** 2006-10-11

**Authors:** Vinod Scaria, Manoj Hariharan, Souvik Maiti, Beena Pillai, Samir K Brahmachari

**Affiliations:** 1 GN Ramachandran Knowledge Center for Genome Informatics, Institute of Genomics and Integrative Biology, CSIR, Mall Road, Delhi 110 007, India

## Abstract

MicroRNAs (miRNAs) are a new class of 18–23 nucleotide long non-coding RNAs that play critical roles in a wide spectrum of biological processes. Recent reports also throw light into the role of microRNAs as critical effectors in the intricate host-pathogen interaction networks. Evidence suggests that both virus and hosts encode microRNAs. The exclusive dependence of viruses on the host cellular machinery for their propagation and survival also make them highly susceptible to the vagaries of the cellular environment like small RNA mediated interference. It also gives the virus an opportunity to fight and/or modulate the host to suite its needs. Thus the range of interactions possible through miRNA-mRNA cross-talk at the host-pathogen interface is large. These interactions can be further fine-tuned in the host by changes in gene expression, mutations and polymorphisms. In the pathogen, the high rate of mutations adds to the complexity of the interaction network. Though evidence regarding microRNA mediated cross-talk in viral infections is just emerging, it offers an immense opportunity not only to understand the intricacies of host-pathogen interactions, and possible explanations to viral tropism, latency and oncogenesis, but also to develop novel biomarkers and therapeutics.

## Background

MicroRNAs (miRNAs) are small RNA molecules which have recently gained widespread attention as critical regulators in complex gene regulatory networks in eukaryotes. These small RNA, processed from non-coding regions of the genome into 18–23 nucleotide long single stranded RNA, have been shown to regulate translation of messenger RNA (mRNA) by binding to it and effecting target cleavage or translational block depending on the extent of sequence complementarity with the target [1]. Generally, in mammalian systems, microRNAs bind to targets with incomplete complementarity, in association with a host of cellular proteins – what is commonly termed as the RNA Induced Silencing Complex (RISC).

MicroRNA mediated regulation has been lately shown to encompass a wide spectrum of host biological processes ranging from growth and development to oncogenesis [2-5]. Recent genome-wide computational screens for microRNA targets in humans predict that 10% [6] to 30% [7] of all genes are regulated by microRNAs. The regulatory network of miRNA-mRNA interaction is rendered even more complex because of multiplicity and cooperativity of microRNA targeting.

MicroRNAs have recently been implicated in the intricate cross-talk between the host and the pathogen [8] in viral infections and is thought to play a major role in viral pathogenesis. Though studies into the entire spectrum of host-pathogen interactions at the microRNA level are still in its infancy, there has been a recent spurt in reports exploring the possibility in a number of major pathogenic viruses of humans.

MicroRNAs were initially discovered in 1993, in a genetic screen for mutants that disrupt the timing of post-embryonic development in the nematode *Caenorhabditis elegans *[9], and were thought to be an oddity in gene regulation, of nematodes till 2000. About 7 years later, when let7 was discovered [10], and was found to be highly conserved in eukaryotes, it led to a surge in discovery of new microRNAs in a number of organisms including humans.

## Biogenesis and mechanism of action of microRNAs

### MicroRNA gene location

MicroRNAs have been classically thought to be transcribed from intergenic regions, but recent large-scale genome-wide cloning experiments [11] have shown that microRNAs can be derived from introns as well. Intergenic microRNAs are sometimes found to occur as clusters which would be transcribed as polycistronic transcripts and are shown to share similar expression profiles [12]. A significant proportion of microRNAs are encoded within the introns of protein-coding genes, presumably expressed in sync with them. A few microRNAs have been mapped to the exons of protein-coding genes. One example is hsa-mir-20a which is annotated by miRBase to arise from the exon 2 as well as introns 5 and 8 of alternative transcripts of C13orf25. The significance of these microRNAs and their roles in alternatively spliced transcripts are yet to be addressed.

### Maturation

MicroRNAs are transcribed by RNA Polymerase II as primary microRNAs (pri-miRNAs) which range from hundreds to thousands of nucleotides in length and resemble protein-coding transcripts in that they are poly-adenylated and capped [1]. These pri-miRNAs are then processed by nuclear localized enzymes Drosha or Pasha (DGCR8 in humans) to produce thermodynamically stable hairpin structures known as pre-microRNAs (pre-miRNAs), of ~70 bases. These pre-microRNAs are then exported to the cytoplasm by Exportin-5 and are then further processed by the RNAase III enzyme Dicer, to form duplexes of 18–23 bases. This duplex is unwound by a yet to be discovered helicase. In steps shared with the siRNA (small interfering RNA) pathway the strand with lower stability in the 5' end (guide strand) is preferentially selected [13] from the double stranded molecule (which composes of miRNA and its complementary strand miRNA*) to be associated with the RNA Induced Silencing Complex (RISC).

### Mechanisms of action

The mechanism of action of microRNAs is considered to be by two modes – translational repression and target degradation. The former is common in mammalian systems while the latter is found predominantly in plants. The basic difference in the two mechanisms is thought to be primarily governed by the levels of complementarity between microRNAs and their target transcripts. Perfect or near perfect complementarity as is common in plant microRNAs and in a small class of eukaryotic microRNAs causes target cleavage and degradation [14], analogous to the action of siRNAs which have perfect complementarity to the target regions. Evidence suggests that microRNA bound transcripts are sequestrated into P bodies [15,16] where they are maintained in a silenced state either by associating with proteins that prevent translation or possibly by removal of the cap structure [16].

**Figure 1:**
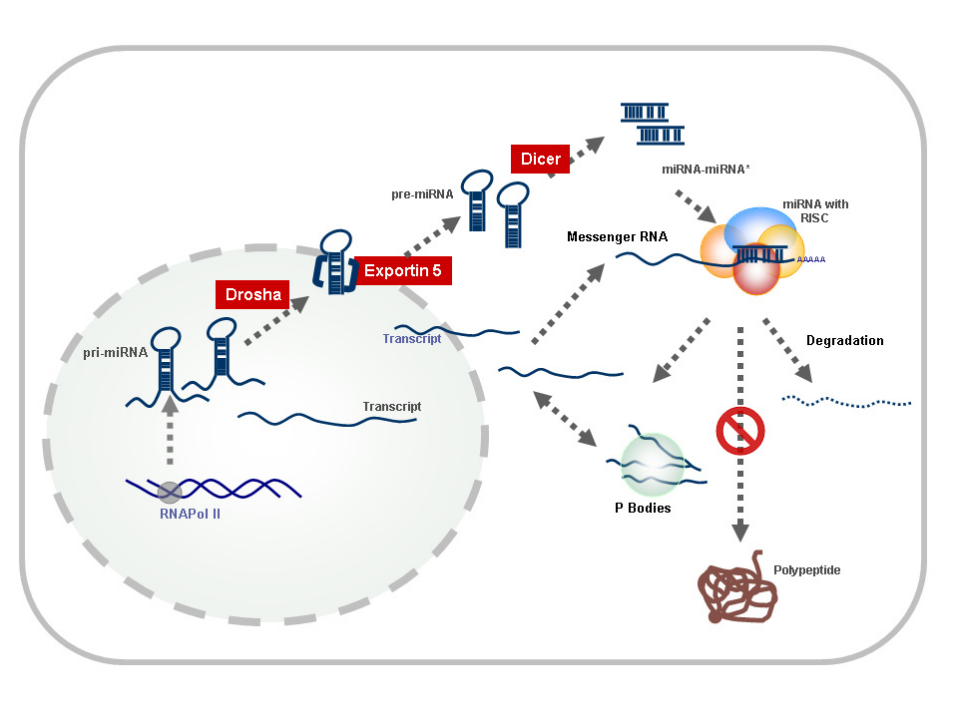
Schematic overview of biogenesis and action of microRNAs in eukaryotic cells.

In fact, one microRNA can have binding sites in multiple targets (in humans, this comes to hundreds) and one target can be repressed by multiple microRNAs (multiplicity and co-operativity) [6]. Moreover, the target repression is likely to be dosage dependent, adding to the complexity of the network of genetic regulation. Recent evidence also suggests an overdose of artificial short hairpin RNAs (shRNAs) can saturate the RNA interference machinery [17]. This would have far reaching implications on determining dosage of artificial microRNAs for therapeutics as well as for experimental research. The biogenesis and action is summarized in Figure 1.

### Non-classical mechanisms of action

Recent evidence suggests that microRNAs can also regulate protein expression through non-classical ways. Genome-wide expression analysis of microRNA targets have shown to decrease the transcript levels [18]. But whether this is a direct or indirect effect is yet to be explored. A couple of recent reports also suggest that microRNAs can modulate de-adenylation of transcripts [19,20]. MicroRNAs are classically thought to be negative regulators, but with exceptions as in the case of a reported human microRNA which can cause abundance of Hepatitis RNA [21] (*vide infra*). A recent report by Bhattacharyya *et al *suggests that microRNA mediated mechanism of post-transcriptional repression of gene expression is indeed reversible [22], suggesting that the proteins associated with microRNA-mRNA complex modulate the expression of the transcript in a switch-like mechanism, responding to particular stimuli. This mechanism, if proved to be a generalized phenomenon, would have large implications in the regulation of gene expression in relation to particular stimuli. In summary microRNAs can act as a trans-acting element for reversible and dynamic regulation of spatial and temporal protein expression.

## Computational tools for discovery of microRNA and their targets

Computational predictions have been the mainstay for discovery of microRNAs and their targets. The algorithms for microRNA prediction range from custom-made programs to search for hairpin loops and energetic stability to advanced algorithms employing machine learning approaches [23-25]. Even though the algorithms for microRNA prediction have improved over time, accurate *de novo *prediction of microRNA still remains a challenging task. This is especially important in the case of viruses as viral microRNAs do not share close homology even in the same class. We hope the prediction algorithms will improve with a better understanding of the sequence and structural components of precursor hairpins involved in microRNA biogenesis [26]. We have recently developed a *de novo *method (unpublished results) for microRNA prediction in viruses based on Support Vector Machines, rationalizing that virus encoded microRNA precursor hairpins would share the sequence and structure features with that of host as they share the same microRNA processing machinery. The algorithms for microRNA target prediction also have been significantly improved from first-generation algorithms which rely on sequence complementarity rules, thermodynamic stability and conservation [6,27,28] by incorporation of features like target RNA structure [29].

## MicroRNAs as an antiviral defense mechanism

Viruses are obligate intracellular parasites and use the cellular machinery for their survival and replication. The success of the virus essentially depends on its ability to effectively and efficiently use the host machinery to propagate itself. This dependence on the host also makes it susceptible to the host gene-regulatory mechanisms. Though the gene regulatory mechanisms involving both host and viral proteins have been extensively studied, data on small RNA mediated gene regulation in viral infections is just emerging. Interestingly, it seems that the cellular microRNAs, in addition to their normal regulatory roles in cellular gene expression also double up as fortuitous agents that target foreign nucleic acids, as in the case of viruses. The inventory of microRNAs encoded differs between cell types, and thus may contribute to the tissue tropism of viruses. Some of these are elaborated in the subsequent sections.

### Primate Foamy Virus

Lecellier *et al *[30], for the first time demonstrated that a mammalian microRNA, mir-32 restricts the accumulation of the retrovirus primate foamy virus type 1 (PFV-1) in human cells. PFV is a retro-transcribing virus similar to the Human Immunodeficiency Virus (HIV), but codes for two additional proteins, Tas and Bet. Insights into the possible role of microRNAs came from the observation that cell lines which express a protein, which interferes with the RNA mediated silencing machinery, showed higher accumulation of PFV-1. Disruption of the target site in a mutant of PFV allowed it to accumulate much faster than the wild type, in infected cells. The group also demonstrated that Tas could act as a non-specific suppressor of RNA interference and could demonstrate that mir-32 related translational block was indeed higher in Tas(-) cells where Tas was not expressed. This report not only throws light into the role of microRNAs in antiviral defense, but also into how viruses have evolved to offset the effects of RNA interference by encoding suppressors of interference.

### Human Immunodeficiency Virus

We have earlier shown [31], using robust computational tools, that involve consensus prediction approaches that five human encoded microRNAs can potentially target the entire repertoire of accessory genes in HIV, including *nef*. The targets were found to be highly conserved in all of the viral clade sequences with the exception of clade O. The fact that defective *nef *is well known to be associated with a long term non-progressor state led us to speculate that the levels of the cellular microRNAs would be a decisive factor in determining the progression of the disease. The targets have been experimentally validated (unpublished results) by cloning the target site in the 3'UTR of Green Fluorescent Protein (GFP) reporter gene. Analysis of previously reported microarray data [32] of these microRNAs in T Cells, demonstrated that the microRNA levels are indeed variable among individuals. Although it is believed that HIV encodes for suppressors of RNAi [33] (vide infra), recent microarray data on microRNA gene expression levels in HIV infected human cells [34] show that above five human encoded microRNAs are down regulated.

### Influenza virus

Through computational methods incorporating both consensus prediction and target accessibility, we have found that human encoded microRNAs could target critical genes involved in the pathogenesis and tropism of Influenza virus A/H5N1 (unpublished results). Two human encoded microRNAs mir-507 and mir-136 had potential binding sites in Polymerase B2 (PB2) and Hemagglutinin (HA) genes respectively. The target regions in the respective genes were not only found to be conserved across different viral strains, but were also found to fall in highly accessible regions of the predicted target RNA structure. Moreover, analysis of previously reported [35] microarray data on microRNA gene expression in different tissues has shown that mir-136 is expressed in lung.

Both the genes PB2 and HA are known to be critical for the pathogenicity of the virus. While HA is the surface glycoprotein involved in direct binding of the virus to the cell surface, HA in the H5N1 subtype carries a polybasic site, cleavage at which, by cellular proteases is an essential step in establishing infection. PB2 is one of the three components of the Ribonucleoprotein which is responsible for RNA replication and transcription. Recent evidence, from recombinant viruses generated by combinations of murine and avian viruses identified PB2 as one of the two genes associated with virulence. The polymerase activity was directly correlated with the high virulence of the murine strain in their cognate host [36]. Another interesting feature is that these microRNAs were found to be absent in the chicken genome, although a large number of human microRNAs (160 of 336 human microRNAs) have homologs in the chicken genome implicating them in the difference in infectivity and lethality of the virus in chicken and human.

## Mammalian microRNAs as positive regulators

### Hepatitis C virus

Jopling *et al *[21] reported an interesting case wherein the tissue specificity of microRNA expression was exploited by a virus to establish tissue selectivity. A liver specific microRNA mir-122 was shown to cause accumulation of viral RNA by binding to the 5' non-coding region of the viral genome. The authors have also verified the findings by mutational analysis as well as sequestration of the microRNA. It is possible that this novel mechanism of microRNAs targeting the 5'UTR of the transcript may be mediated through a translation controlling switch at the 5'end of the transcript through changes in RNA secondary structure. This, by and large, remains the only report of a microRNA targeting the 5'UTR in a mammalian system and causing RNA accumulation.

## Viral suppressors of RNAi mediated gene silencing

Interestingly viruses have also evolved to evade RNAi by a variety of strategies. The suppressors of antiviral RNAi is better understood in plant viruses. To counteract the small RNA mediated interference, viruses express suppressors that interfere with microRNA as well as siRNA pathways [37]. The virus encoded antiviral RNAi suppressors include small RNAs to proteins, and are thought to effect through various mechanisms like sequestration and/or inhibition of siRNA formation [38-40]. The entire spectrum of suppressors of RNAi encoded by animal viruses is yet to be unraveled.

## Virus encoded microRNAs

The interest in discovering novel microRNA candidates using both computational tools and experimental validation of the predicted candidates have shown that viruses also encode microRNAs (see Table 1). Most of these predictions have been successfully validated using experimental approaches. The current understanding of virus encoded microRNAs is limited mainly to the Herpes virus family, which is a unique class of viruses whose members are implicated in a number of major pathogenic states in humans ranging from mild infections to oncogenesis. Other viruses with miRNA mediated regulation include major pathogens like HIV and Simian Virus 40 (SV40) [41]. SV40 encoded microRNAs which are generated during the late phase in life cycle could target the early transcripts including those coding for viral T antigens and mediate evasion of the virus infected cells from cytotoxic T cells.

**Table 1:** List of virus-encoded microRNAs

**Source Virus**	**Virus Type**	**Number of microRNAs**	**References**
Epstein Barr virus	Herpesvirus	32	[42]
Kaposi sarcoma-associated herpesvirus	Herpesvirus	17	[43]
Mouse gammaherpesvirus	Herpesvirus	10	[42]
Human cytomegalovirus	Herpesvirus	14	[42]
Herpes Simplex-1	Herpesvirus	1	[44]
Rhesus lymphocryptovirus	Herpesvirus	22	[45]
Simian virus 40	Papovavirus	2	[41]
Human Immunodeficiency Virus	Retrovirus	1	[46]

Interestingly the viral microRNAs, unlike their vertebrate counterparts do not share a high level of homology, even within members of the same family or with that of the host. This has been attributed to the higher rate of mutations and the faster evolution in viruses as compared to eukaryotes. Though this would mean an evolutionary advantage to rapidly adapt to the host and environmental conditions, it offers a challenge to computational biologists as most of the algorithms for microRNA prediction relies heavily on conservation and would prove inadequate in case of viruses. This would be just the tip of the iceberg as *de novo *prediction of microRNA candidates is still an unmet challenge for computational biologists. With the advent of high throughput validation methods like microarrays being employed, and with better and efficient computational algorithms for prediction of microRNAs, the count is all set to rise. Similarly there is a severe gap in the understanding the targets of these microRNAs and necessitates the use of better technology clubbed with efficient computational algorithms.

### Regulation of cellular processes by virus encoded microRNA

Bennasser *et al *[33] reported a computational screen for HIV-1 encoded microRNAs and further went about predicting their cellular targets and found five pre-miRNA candidates which has potential to encode 10 microRNAs and through them regulate ~1000 host transcripts. In a similar computational screen for targets to potential HIV encoded microRNA, Couturier *et al *[47] showed that the HIV pro-viral genome had multiple matches of complementarity in important cellular proteins/cytokines well known to play crucial roles in HIV pathogenesis like CD28, CD40L, IL-2, IL-3, TNF-β, IL-12 and CD4. The current understanding is that multiple gapped stretches of complementarity can result in translational repression, along with the discovery that the HIV-1 pro-viral genome has such levels of complementarity especially with human protein coding genes, points to the possibility that HIV transcripts may encode for microRNAs, or small regulatory RNAs. This along with the evidence that a large number of these cellular genes including CD28 are well documented to be down regulated in HIV pathogenesis, points to the possible cross-talk between the virus and host at the microRNA level.

Of late, Cui *et al *discovered novel virus encoded microRNAs from HSV genome [44]. Subsequently Gupta *et al*, discovered that Herpes simplex-1 (HSV-1) latency associated transcript (LAT) encodes for a microRNA which target critical genes of the apoptosis pathway [48] including those involved in the TGF-β signaling pathway, including TGF-β1 and SMAD3, thereby protecting the cells from apoptosis. This is perhaps the first experimental evidence of a virus encoded microRNA targeting cellular transcripts.

Such a mechanism could operate in other related herpes viruses like Epstein-Barr virus, which not only causes latent infection, but also associated with a wide spectrum of neoplasms in humans including Burkitts lymphoma and nasopharyngeal carcinoma. It is all the more probable that oncogenic herpes viruses like EBV and KSHV encoded microRNAs can target critical genes involved in oncogenesis. This is especially so in the case of EBV encoded microRNAs as they have been shown to be differentially expressed in different phases of the viral life [45]. Our analysis of cellular targets of 32 EBV encoded microRNAs using robust computational approaches using consensus of microRNA target prediction software revealed that the target genes are involved in apoptosis and tumor suppressor pathways, suggesting that EBV encoded microRNAs play crucial roles in oncogenic transformation induced by the virus (unpublished results). The discovery of virus encoded microRNAs playing crucial roles in pathogenesis of diseases caused by viruses not only throws light on a new level of host-pathogen interactions, but also would help in designing novel preventive and therapeutic strategies.

### Viral gene regulation by virus-encoded microRNAs

Omoto *et al*, using a combinatorial approach incorporating both computational prediction and experimental validation demonstrated the possibility that a virus-encoded microRNA could auto-regulate itself. A *nef *derived microRNA could down regulate *nef *expression in vitro suggesting that it could be a mechanism of maintaining low viremia in Long term non-progressor (LTNP) states [46]. This finding was later expanded by the same group with an additional discovery that *nef *derived microRNA also suppress transcription [49] by reducing HIV-1 promoter activity through the negative responsive element in the 5'-LTR, thus contributing to an additional layer of auto regulation.

In yet another instance, a virus encoded microRNA, is effectively used by the virus to tune down a set of genes and thereby evade cytotoxic T cell response [41]. Simian Virus 40 (SV40) was shown to produce a microRNA which was primarily expressed in the late stages of infection. Curiously, the expression of the viral microRNAs did not have any untoward effect on the viral replication. Further analysis revealed that the microRNAs had near perfect complementary matches in the early expressed genes of the virus which would target them for interference mediated degradation. The genes include the T antigen which is a determinant for invasion of T cells, thus providing an advantage in camouflaging the virus infected cells from the cellular immune system.

## MicroRNAs as biomarkers and therapeutics

Recently microRNA expression profiles have been analyzed for viral infections like HIV [34]. MicroRNA expression has been shown to be specific to various stages of infection in Herpesviruses [45] and have been proposed to be associated with latency in HIV infection [31,50], promising an early biomarker for cancers caused by oncogenic viruses. MicroRNA profiles have also been explored in a number of patho-physiological conditions [51]. Recent reports suggest that microRNA profiles can be used not only to classify different classes of cancers [52-54], but could also be used as biomarkers for diagnosis and prognosis of disease states [54].

MicroRNAs and anti-microRNA oligonucleotides (AMOs) have been proposed as novel therapeutics [55,56]. Recent advances in nucleotide chemistry like Locked Nucleic Acids (LNA) [57], and other backbone modifications have made it possible to design small RNA oligonucleotides which are highly stable in biological systems circumventing one major hurdle in using microRNAs as future therapeutics. Oligonucleotide modifications have already made their way to the microRNA experimental biologists workbench [58]. The success of siRNA based strategies in targeting specific genes could be extended to microRNAs also. This would include delivery strategies [34]. This would be even more important as siRNA based therapeutics for viral pathogens in different stages of clinical trials and are showing promising results.

### Artificial microRNAs (amiRNAs) and microRNA engineering

MicroRNAs are promising candidates for developing novel bio-therapeutics against viruses, as it requires only partial complementarity unlike siRNAs and thus can tackle the high rate of mutations in viruses better than siRNAs. Initial experiments creating microRNAs based on siRNA design rules have shown promising results [59]. Our group has recently developed an algorithm for design of highly specific microRNAs on against sequences to be targeted (unpublished results). This would allow design of microRNAs against highly conserved sequences in viral genome. Artificial microRNAs also offer the advantage that they can be optimized to create less off-target events in the host thus substantially reducing untoward side effects. MicroRNAs or microRNA target sequences could also be engineered into transforming viruses and could enable tissue specific and environment sensitive expression of genes.

## Integrative approach to modeling microRNA mediated host-virus interaction

**Figure 2:**
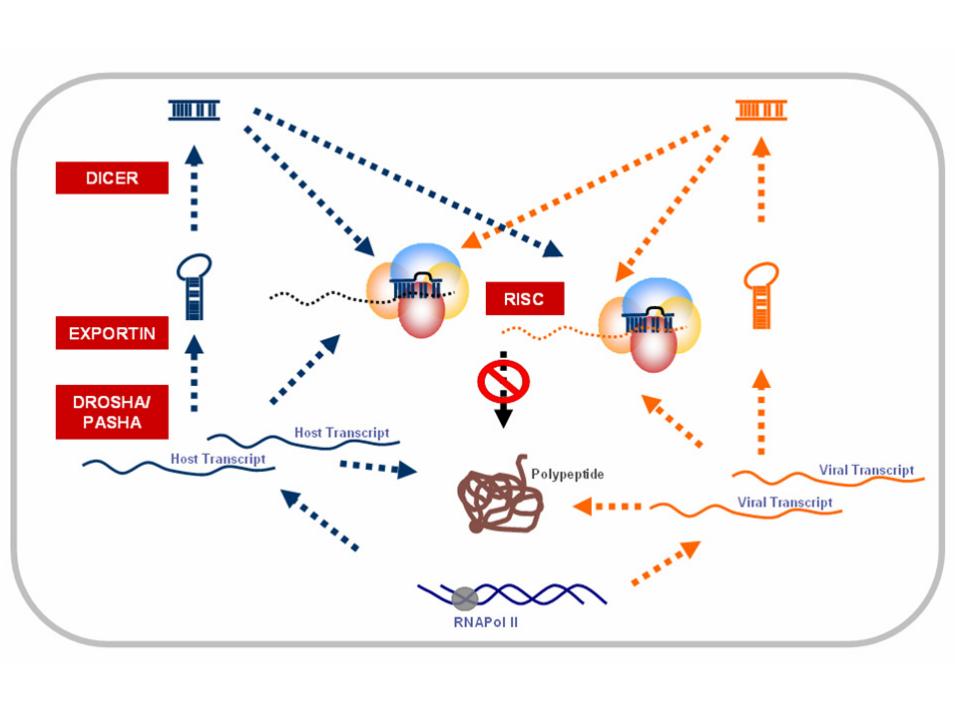
Complexity of microRNA mediated host-pathogen Interaction. The Enzymes in the Biosynthesis of microRNAs are highlighted in RED boxes. The viral transcripts and products are colored in ORANGE, while those of host are colored in BLUE.

With evidence demonstrating that both host encoded and virus encoded microRNAs interact with host and virus transcripts respectively, in addition to their roles in regulating their own transcripts warrants a comprehensive analysis of host and virus microRNAs and their targets to elucidate a holistic picture of microRNA mediated host-virus interaction model (Figure 2). The challenge would be to integrate bioinformatics with gene expression and proteomics data. This would not only enable them to design novel diagnostic and therapeutic strategies to combat deadly viruses, but also empower researchers to understand basic biological processes involved in latency and oncogenic transformation mediated by viruses.
